# FKBP12 is a major regulator of ALK2 activity in multiple myeloma cells

**DOI:** 10.1186/s12964-022-01033-9

**Published:** 2023-01-30

**Authors:** Ingrid Quist-Løkken, Clara Andersson-Rusch, Martin Haugrud Kastnes, Jürgen Markus Kolos, Jerome Jatzlau, Hanne Hella, Oddrun Elise Olsen, Anders Sundan, Petra Knaus, Felix Hausch, Toril Holien

**Affiliations:** 1 Department of Clinical and Molecular Medicine, Norwegian University of Science and Technology - NTNU, Trondheim, Norway; 2 Centre of Molecular Inflammation Research, Norwegian University of Science and Technology - NTNU, Trondheim, Norway; 3 Department of Chemistry, Technical University of Darmstadt, Darmstadt, Germany; 4 Institute for Chemistry and Biochemistry, Freie Universität Berlin, Berlin, Germany; 5 Department of Immunology and Transfusion Medicine, St. Olav’s University Hospital, Trondheim, Norway; 6 Department of Hematology, St. Olav’s University Hospital, Trondheim, Norway; 7 Department of Biomedical Laboratory Science, Norwegian University of Science and Technology - NTNU, Trondheim, Norway

**Keywords:** Hematological cancer, SMAD, BMP, ACVR1, BMPRII, Tacrolimus, FKBP

## Abstract

**Background:**

The immunophilin FKBP12 binds to TGF-β family type I receptors, including the BMP type I receptor ALK2. FKBP12 keeps the type I receptor in an inactive state and controls signaling activity. Removal of FKBP12 with drugs such as the FKBP-ligand FK506 enhances BMP activity in various cell types. In multiple myeloma cells, activation of SMAD1/5/8 leads to apoptosis. We hypothesized that removing FKBP12 from ALK2 in myeloma cells would potentiate BMP-induced ALK2-SMAD1/5/8 activity and in consequence cell death.

**Methods:**

Multiple myeloma cell lines were treated with FK506, or other FKBP-binding compounds, combined with different BMPs before analyzing SMAD1/5/8 activity and cell viability. SMAD1/5/8 activity was also investigated using a reporter cell line, INA-6 BRE-luc. To characterize the functional signaling receptor complex, we genetically manipulated receptor expression by siRNA, shRNA and CRISPR/Cas9 technology.

**Results:**

FK506 potentiated BMP-induced SMAD1/5/8 activation and apoptosis in multiple myeloma cell lines. By using FKBP-binding compounds with different affinity profiles, and siRNA targeting FKBP12, we show that the FK506 effect is mediated by binding to FKBP12. Ligands that typically signal via ALK3 in myeloma cells, BMP2, BMP4, and BMP10, did not induce apoptosis in cells lacking ALK3. Notably, BMP10 competed with BMP6 and BMP9 and antagonized their activity via ALK2. However, upon addition of FK506, we saw a surprising shift in specificity, as the ALK3 ligands gained the ability to signal via ALK2 and induce apoptosis. This indicates that the receptor complex can switch from an inactive non-signaling complex (NSC) to an active one by adding FK506. This gain of activity was also seen in other cell types, indicating that the observed effects have broader relevance. BMP2, BMP4 and BMP10 depended on BMPR2 as type II receptor to signal, which contrasts with BMP6 and BMP9, that activate ALK2 more potently when BMPR2 is knocked down.

**Conclusions:**

In summary, our data suggest that FKBP12 is a major regulator of ALK2 activity in multiple myeloma cells, partly by switching an NSC into an active signaling complex. FKBP12 targeting compounds devoid of immunosuppressing activity could have potential in novel treatment strategies aiming at reducing multiple myeloma tumor load.

**Video Abstract**

**Supplementary Information:**

The online version contains supplementary material available at 10.1186/s12964-022-01033-9.

## Background

Bone morphogenetic proteins (BMPs) is a subgroup of the transforming growth factor (TGF)-β family [1]. BMPs signal by binding to a complex consisting of two type I and two type II receptors that can be combined in different ways. In the TGF-β pathway, two different SMAD branches can be activated depending on the type I receptor. When the BMP type I receptors ALK1, ALK2, ALK3 or ALK6 are part of the active signaling complex, the SMAD1/5/8 pathway is activated, while if the TGF-β/activin type I receptors ALK4, ALK5 or ALK7 are present, the SMAD2/3 pathway is activated [2]. ALK2 and ALK3 are the most widely expressed BMP type I receptors, that, dependent on cellular context, can act as receptors for a wide range of BMPs [3]. There are three BMP type II receptors, BMPR2, ACVR2A and ACVR2B, which are shared with other TGF-β ligands, such as the activins and growth differentiation factors (GDFs) [3]. The receptor promiscuity makes ligands compete for binding to receptors/receptor complexes and has a huge impact on the signaling outcome [3, 4]. Notably, non-signaling complexes (NSC) have been described where activin A binds ALK2:type II receptor complexes making both the ligand and the receptors unavailable for signaling by other ligands, such as BMPs [5–8]. Fibrodysplasia ossificans progressiva (FOP) is a genetic connective tissue disease in which mutations in *ACVR1*, encoding ALK2, make activin A switch from a competitor of signaling (e.g. NSC) to an agonist [6]. The idea that small changes can switch NSCs into active signaling complexes brings variability to the system and represents a novel way of looking at ligand-receptor complexes in the TGF-β family.

Other molecules also bind the ligand-receptor complexes and regulate their activity. The intracellular FK506-binding protein 12 (FKBP12) is one such molecule which was discovered in the 1980s as a receptor for the immunosuppressant drug FK506 [9, 10]. FK506, also known as Tacrolimus, was first discovered in Japan in 1987 [11]. Its immunosuppressant effect was described in 1991 [12], and in 1994 it was approved for use in liver transplant patients [13]. From then on FK506 has been used clinically as an immunosuppressant drug after organ transplantations. FK506 binds the 15 human FKBPs with different affinities, but the immunosuppressing effect is mediated by binding and forming a complex with FKBP12 that inhibits calcineurin in immune cells, such as T-lymphocytes [14, 15]. Cyclophilins is another group of proteins that form calcineurin-inhibiting drug-complexes together with the drug cyclosporin A (CsA). Collectively, FKBPs and cyclophilins are therefore termed immunophilins [16]. Many immunophilins catalyze the *cis*/*trans* isomerization of peptidyl-prolyl reactions but this is not their only function [17]. One described function of FKBP12 is that it binds to the intracellular glycine-serine-rich (GS) domain of ligand-free TGF-β type I receptors, including the BMP type I receptors, and inhibits their activation by type II receptors [18–20]. When FK506 is present it competes with the type I receptor for binding of FKBP12 in vitro and in vivo [18, 21]. When FKBP12 is removed from the TGF-β signaling complex, e.g. by binding to FK506, the result is unrestricted SMAD-signaling and enhanced ligand-induced SMAD-activation [20, 22, 23]. FK506 can therefore be used to regulate SMAD activity.

Multiple myeloma is a cancer of the bone marrow which arises from plasma cells. The disease is considered incurable as the myeloma cells over time become resistant to all current therapies, including new immune- and CAR-T cell therapies [24, 25]. Many BMPs can induce growth arrest and apoptosis of myeloma cells in vitro [26–30] and BMP4 gene therapy inhibited tumor growth in a multiple myeloma mouse model [31]. The relevant BMP type I receptors in this context are ALK2, ALK3, and to some extent ALK6, whereas ALK1 is not expressed in multiple myeloma cells [30]. We here show that FK506 potentiated BMP-induced SMAD1/5/8-activation and apoptosis in multiple myeloma cells, likely by sequestering FKBP12. To differentiate between effects on different ligand-receptor combinations, we took advantage of cells expressing the BMP-type I receptor ALK2, but little or no expression of ALK3. These cells responded poorly to the ligands BMP2, BMP4 and BMP10, that normally would require ALK3 to signal in myeloma cells [28, 30]. Notably, even though BMP10 was not signaling it competed with BMP6 and BMP9 and antagonized their activity via ALK2, indicating that the ligand could bind to BMP receptor complexes on these cells. Surprisingly, we saw a switch in activity from an inactive NSC to an active ligand-receptor complex, as BMP2, BMP4, and BMP10 gained the ability to signal via ALK2 when FKBP12 was sequestered with FK506. Furthermore, unlike BMP6 and other common ALK2 ligands, BMP2, BMP4 and BMP10 need BMPR2 for full activity via ALK2. The gain-of-function towards ALK2 was also seen in other cell types with similar receptor expression patterns, indicating that the observed effects have broader relevance. In summary, our results suggest that FKBP12 is a major regulator of ALK2 activity in myeloma cells, partly by switching an NSC into an active signaling complex.

## Methods

### Cells and reagents

The human myeloma cell lines used here were INA-6 (a kind gift from Dr Martin Gramatzki, University of Erlangen-Nurnberg, Erlangen, Germany) [32], IH-1 [27] and KJON [33] (established in our lab), and KARPAS-417 (ECACC, #06100302). We also used the stomach cancer cell line KATO-III (ATCC), and the B-cell lymphoma cell line DOHH-2 (DSMZ). INA-6 were grown in 10% heat-inactivated fetal calf serum (FCS) in RPMI-1640 (Sigma-Aldrich, Oslo, Norway) supplemented with 2 mM l-glutamine (Sigma-Aldrich) (RPMI) and interleukin (IL)-6 (1 ng/mL) (Gibco, Thermo Fisher Scientific, Waltham, MA, USA). IH-1 cells were cultured using 10% heat-inactivated human serum (HS) (Department of Immunology and Transfusion Medicine, St. Olav’s University hospital, Trondheim, Norway) in RPMI and IL-6 (2 ng/mL). KJON cells were grown in 5% HS in RPMI with IL-6 (2 ng/mL). KARPAS-417 and DOHH-2 were grown in 10% FCS in RPMI, and KATO-III was grown in 20% FCS in RPMI. Stably transduced INA-6 shCTR and shBMPR2 cells were described before and grown as regular INA-6 cells [34]. All cell lines were regularly checked for mycoplasma. Recombinant human BMP2 (#355-BM), BMP4 (#314-BP), BMP6 (#507-BP), BMP9 (#3209-BP), BMP10 (#2926-BP), TGF-β1 (#240-B), and activin B (#659-AB-025) were from R&D Systems (Bio-Techne, Abingdon, UK). Human recombinant activin A was produced in *E. coli* and kindly provided by Dr. Marko Hyvönen’s group at the University of Cambridge, UK [35]. The chemical inhibitor FK506 was from Selleck Chemicals (Munich, Germany), whereas K02288, SB431542, cyclosporin A (CsA), and rapamycin (RAP) were from Sigma-Aldrich. SAFit1, and the compounds 19 and 19^(S)-Me^ were synthesized as described before [36, 37]. Compound 19 [37] is structurally identical to compound 16j [36].

### Cell viability and proliferation

CellTiter-Glo (Promega, Madison, WI, USA) measures ATP levels in cells by luciferase and was used to measure relative cell proliferation. Cells were seeded in 2% HS in RPMI in 96 well plates and were treated as indicated. CellTiter-Glo was added to the cells, the plate was then shaken for 2 min before 10 min incubation at room temperature and reading of the plate using Victor 1420 multilabel counter (PerkinElmer Inc. Waltham, MA, USA).

### Caspase-3/7 activity

To measure caspase-3/7 activity, 10 000 cells were seeded per well in 96 well plates in 2% HS in RPMI and treated as indicated for 48 h. Caspase-Glo 3/7 Reagent (Promega, # G8091) was added and the plate was shaken for 2 min and incubated for 30 min at RT before recording luminescence with Victor 1420 multilabel counter.

### Apoptosis assay

To measure cell viability, cells were seeded in 2% HS in RPMI and treated as indicated before they were stained using Apoptest Annexin A5-FITC kit (VPS Diagnostics, Hoeven, The Netherlands). In brief, cells were incubated with annexin V-FITC (0.2 µg/mL in binding buffer) for 1 h on ice. Propidium iodide (PI) (1.4 µg/mL) was added 5 min before data acquisition using an LSRII flow cytometer (BD Biosciences, San Jose, CA). Cells negative for both annexin V and PI staining were counted as viable.

### BRE-luc reporter assays

INA-6 cells were transduced with lentivirus to insert a BMP responsive element, driving the expression of luciferase and the construct was described previously [37]. The BMP responsive element was derived from a mouse *Id1* promoter, and is activated by SMADs after stimulation with BMPs [38]. The cells were washed with Hanks balanced salt solution and resuspended in 0.1% bovine serum albumin (BSA) in RPMI with IL-6 (1 ng/mL) as experimental media. The cells were seeded out in a 96-well plate, with 50 000 cells/well and stimulated overnight (18 h) as indicated. After treatment BriteLite™ plus luciferase detection reagent (PerkinElmer Inc.) was added according to manufacturer’s protocol and the luminescence was detected using a Victor 1420 multilabel counter.

### Western Blot

Cells were seeded in 2% HS in RPMI and treated as indicated, washed with ice cold phosphate-buffered saline (PBS) and lysed for 30 min on ice. The lysis buffer contained 1% IGEPAL® CA-630 (Sigma- Aldrich), 10% glycerol, 150 mM NaCl, 50 mM Tris-HCL (pH 7.5), Complete mini protease inhibitor cocktail (Roche, Basel, Switzerland), 1 mM Na_3_VO_4_ and 50 mM NaF. Samples were mixed with LDS sample buffer (Invitrogen, Thermo Fisher Scientific) with 100 mM DTT, heated for 10 min at 70 °C and then separated on NuPAGE Bis-Tris gels (Invitrogen, Thermo Fisher Scientific) followed by wet blotting to 0.45 μm nitrocellulose membranes (Bio-Rad, Hercules, Ca, USA). The membranes were blocked using 5% dry milk in Tris-buffered saline with 0.05% Tween-20 (TBS-T). As primary antibodies we used FKBP12 (RRID: AB_2102847, #sc-133067, Santa Cruz, TX, USA), phospho-SMAD1/5/9 (RRID: AB_2493181, #13820), phospho-SMAD1/5 (RRID: AB_491015, #9516), phospho-SMAD2 (RRID: AB_490941, #3108), and phospho-SMAD2/3 (RRID: AB_2631089, #8828) (Cell Signaling Technology, BioNordika AS, Oslo, Norway), and glyceraldehyde-3-phosphate dehydrogenase (GAPDH) (RRID: AB_2107448, #Ab8245, Abcam, Cambridge, UK). Detection was done using horseradish peroxidase-conjugated secondary antibodies (DAKO Cytomation, Copenhagen, Denmark), SuperSignal West Femto Maximum Sensitivity Substrate (Thermo Fisher Scientific, Waltham, MA, USA) and an Odyssey Fc Imager with Image Studio software (LI-COR Biosciences, Cambridge, UK).

### RT-qPCR

RNA was isolated with RNeasy Mini Kit (Qiagen, Crawley, UK). The cDNA synthesis was performed using High-Capacity RNA-to-cDNA kit (Applied Biosystems, Thermo Fisher Scientific). To perform RT-qPCR, we used standard settings of the StepOne Real-Time PCR system with TaqMan Fast Advanced Master Mix and Taqman Gene Expression Assays, FAM-MGB labelled (Applied Biosystems, Thermo Fisher Scientific). The assays used were *FKBP1A* (Hs00356621_g1), *SMAD1* (Hs00195432_m1), *SMAD5* (Hs00195437_m1), *SMAD4* (Hs00929647_m1), *ACVR1* (Hs00153836_m1), *BMPR1A* (Hs01034909_g1), *BMPR1B* (Hs01010965_m1), *BMPR2* (Hs00176148_m1) and *GAPDH* (Hs99999905_m1). Comparative Ct method was used to calculate the relative changes in expression with GAPDH as housekeeping gene.

### Transfection of siRNA

For transient knockdown experiments INA-6 cells were transfected using the Nucleofector device (Amaxa, Biosystems, Cologne, Germany) and Amaxa Cell Line Nucleofector Kit R (Lonza, Basel, Switzerland) as described previously [39]. We used human ON-TARGETplus SMARTpool FKBP1A (#L-009494-00-0005), ACVR1 (#L-004924-00-0005), BMPR1A (#L-004933-00-0005), SMAD1 (#L-012723-00-0005), SMAD4 (#L-003902-00-0005), SMAD5 (#L-015791-00-0005), and Non-Targeting Pool (#D-001810-10-20) siRNA (Dharmacon, Thermo Fisher Scientific). The siRNA target sequences (each pool of four) are given in Table 1.

**Table 1:** Target sequences of siRNA

Gene name	Target sequences
*FKBP1A*	GAGCCAAACUGACUAUAUC, GACAGAAACAAGCCCUUUA, AAACUGGAAUGACAGGAAU, GAAAUUUGAUUCCUCCCGG
*ACVR1*	GAAUGGACAGUGUUGCAUA, GUCCAUAGCUAGUGGUCUU, GAAAGGCUGCUUCCAGGUU, GUACGACUAUCUUCAGCUU
*BMPR1A*	AAGCAGACGUCGUUACAAU, UGGACUACCUUUAUUGGUU, ACACAUGCAUAACUAAUGG, CAGCUACGCCGGACAAUAG
*SMAD1*	GCUCUAUUGUCUACUAUGA, GGCGGUUGCUUAUGAGGAA, CAACAAUCGUGUGGGUGAA, CAAAUGGGUUCACCUCAUA
*SMAD4*	GCAAUUGAAAGUUUGGUAA, CCCACAACCUUUAGACUGA, GAAUCCAUAUCACUACGAA, GUACAGAGUUACUACUUAG
*SMAD5*	GAUCAGAUGGGUCAAGAUA, CAACUUUCAUCAUGGCUUU, GAGCUAAAGCCGUUGGAUA, GUAGAUGGAUUCACAGAUC
Non-targeting	UGGUUUACAUGUCGACUAA, UGGUUUACAUGUUGUGUGA, UGGUUUACAUGUUUUCUGA, UGGUUUACAUGUUUUCCUA

### CRISPR/Cas9 knock-out of ACVR1

CRISPR sgRNAs targeting *ACVR1* were designed using Benchling (https://www.benchling.com/). Oligo pairs encoding the sgRNA (aaacCGAGACGTGGAGTATGGCAC, CACCGTGCCATACTCCACGTCTCG) were annealed and ligated into lentiCRISPR v2 (gift from Feng Zhang, Addgene plasmid #52961; http://n2t.net/addgene:52961; RRID:Addgene_52961) [40]. As an irrelevant, targeting control, an sgRNA specific for intron 2 of *UNG* was used (sequence CGACCCGCGAGATGATATCA, kind gift from Per Arne Aas, NTNU, Trondheim). The plasmids were cotransfected with third generation lentiviral packaging plasmids using Genejuice (Novagen, Merck Life Science AS, Oslo, Norway) in 293 T packaging cells (Open Biosystems, Thermo Fisher Scientific). The filtered supernatant was used to generate the knockout cells by treating INA-6 with lentiviral particles in the presence of polybrene (8 µg/mL). Fresh medium was added after 4 h. Puromycin (0.5 µg/mL) was added after 48 h to select for cells with successful integration. Cells were seeded as single clones into a 96 well plate. Mutations and loss of function of the INA-6 *ACVR1* K.O. cells were confirmed by blunt-end cloning followed by Sanger sequencing, and functional cell signaling and viability assays.

### Statistical analysis

GraphPad Prism 9 (GraphPad Software, San Diego, LA) was used to analyze statistical significance.

## Results

### FK506 potentiated SMAD1/5/8 activation and apoptosis in multiple myeloma cells

**Fig. 1:**
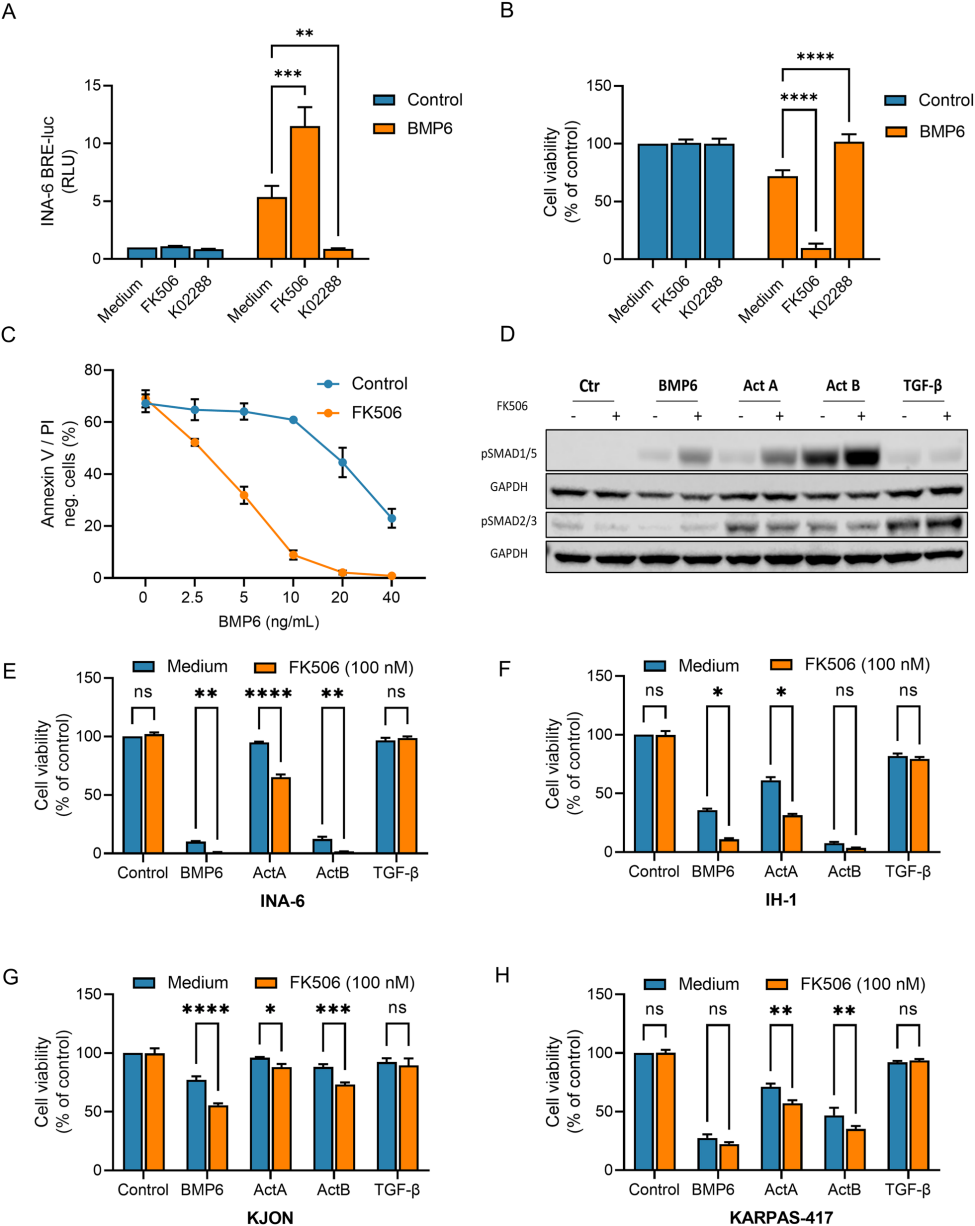
FK506 potentiated SMAD1/5/8 activation and apoptosis in multiple myeloma cells. **A** INA-6 BRE-luc cells were treated for 6 h with BMP6 (5 ng/mL), FK506 (100 nM) or K02288 (100 nM). Luciferase substrate was added, and the results are shown as relative luciferase units (RLU). **B** INA-6 cells were treated with BMP6 (7.5 ng/mL), FK506 (100 nM) or K02288 (100 nM) for 48 h and relative cell viability was measured using CellTiter Glo. The graphs are plotted relative to medium control and each bar represents mean ± standard error of the mean (s.e.m.) of n = 3 independent experiments. Two-way ANOVA and Tukey’s multiple comparisons test was used to analyze statistical significance (*p < 0.05, **p < 0.01, ***p < 0.001, ****p < 0.0001, ns—nonsignificant). **C** INA-6 cells were treated with BMP6 and FK506 for 72 h. The cells were analyzed by annexin V FITC and propidium iodide (PI) staining. Double negative cells were regarded as viable. **D** INA-6 cells were treated with BMP6 (5 ng/mL), FK506 (100 nM), Activin A (50 ng/mL), Activin B (5 ng/mL) and TGF-ß (5 ng/mL) for 2 h before phospho-SMAD1/5/8 was measured by western blotting. GAPDH was used as a loading control. The blot is representative of n = 3 independent experiments. **E**–**H**. The human myeloma cell lines INA-6 (**E**), IH-1 (**F**), KJON (**G**), and KARPAS-417 (**H**) were treated with BMP-6 (50 ng/mL), Activin A (50 ng/mL), Activin B (20 ng/mL), and TGF-β (5 ng/mL), with or without FK506 (100 nM). After 72 h the relative cell viabilities were measured with CellTiter Glo. The graphs are plotted relative to medium control and each bar represents mean ± s.e.m. of n = 3 independent experiments. Two-way ANOVA and Sidak’s multiple comparisons test was used to analyze statistical significance (*p < 0.05, **p < 0.01, ***p < 0.001, ****p < 0.0001, ns—nonsignificant)

Multiple myeloma cells are sensitive to BMP- and activin-induced apoptosis via activated SMAD1/5/8 transcription factors that cause downregulation of the c-MYC oncogene, followed by Bcl-xL downregulation and caspase 3 cleavage [29, 34]. We here aimed to increase myeloma cell death by enhancing SMAD1/5/8 activity. First, we tested the effect of FK506 combined with BMP6 using the reporter cell line INA-6 BRE luc. This cell line stably expresses the BMP-responsive element of the *ID1* promoter in front of luciferase (BRE-luc) [38]. The combination of FK506 with BMP6 strongly potentiated the activation of SMAD1/5/8 in these cells as measured by relative luciferase activity (Fig. 1A). As a negative control we used the BMP type I receptor kinase inhibitor K02288 [41]. We next analysed cell viability by ATP content and as expected FK506 also potentiated the effect of BMP6 in this assay, whereas K02288 blunted it (Fig. 1B). Since this ATP-based cell viability assay does not distinguish between effects on apoptosis and proliferation, we also did dose-response curves of BMP6 with and without FK506 and stained with annexin V and propidium iodide (PI). The effect on apoptosis was dose-dependent and most clear with 10 ng/mL BMP6 (Fig. 1C and Additional file 1: Fig. S1A, for examples of flow cytometry dot plots). We then compared the effect of FK506 on SMAD1/5/8 phosphorylation induced by BMP6, activin A, activin B, and TGF-β (Fig. 1D and Additional file 1: Fig. S1B, C for densitometric analysis). The potentiating effect of FK506 was clear for SMAD1/5/8-activation by BMP6 and the activins, whereas there was no potentiation of activin-induced SMAD2/3 activity. TGF-β-induced SMAD-activation of both branches has been described in this cell line before [42]. TGF-β-induced SMAD-activation was unaffected by FK506 cotreatment in contrast to the activins where SMAD1/5, but not SMAD2/3 activation was potentiated. Having established that FK506 potentiated BMP6-induced SMAD1/5/8-activation and apoptosis in INA-6 cells, we next wanted to see how common this was among myeloma cell lines. We compared the effect of FK506 combined with various TGF-β family ligands on reduction of cell viability in nine different cell lines using a fixed, relatively high concentration for each ligand. Seven of the cell lines analysed were sensitive to at least one of the tested ligands, but in only four of the seven could we see a potentiation of ligand-induced reduction in cell viability (Fig. 1E–H and data not shown). However, as we only measured cell viability, we cannot rule out if FK506 can potentiate BMP-induced SMAD1/5/8-activity in the insensitive cell lines. Taken together, FK506 potentiates BMP- and activin-induced SMAD1/5/8 activation and apoptosis in multiple myeloma cells.

### FK506 potentiated SMAD1/5/8 activation via FKBP12

**Fig. 2:**
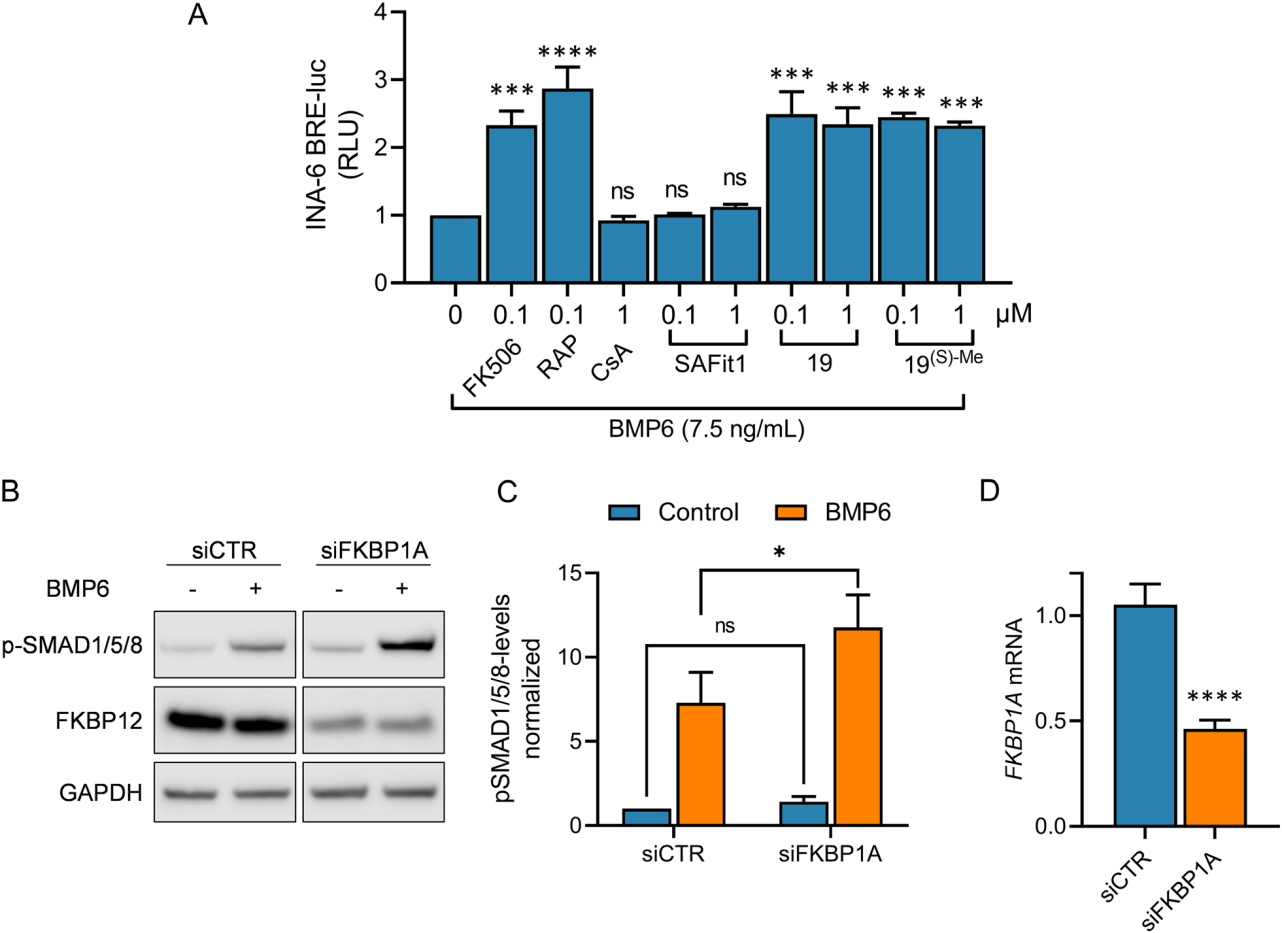
FK506 potentiated SMAD1/5/8 activation via FKBP12. **A** INA-6 BRE-luc cells were treated for 18 h with BMP6 (7.5 ng/mL) and the indicated compounds. Luciferase activity was measured and the results are shown as relative luciferase units (RLU) relative to BMP6. The graphs are plotted relative to medium control and each bar represents mean ± s.e.m. of n = 3 independent experiments. One-way ANOVA and Sidak’s multiple comparisons test were used to analyze statistical significance (*p < 0.05, **p < 0.01, ***p < 0.001, ****p < 0.0001, ns—nonsignificant). **B** INA-6 cells were transfected with FKBP1A or non-targeting control siRNA. Western Blot was then performed on cells treated with and without BMP6 (7.5 ng/mL) for 1 h. The blot is representative of n = 3 independent experiments. **C** Each band obtained from the western blots were quantified to determine the expression of phospho-SMAD1/5/8 and were normalized to GAPDH levels. The graphs are plotted relative to medium control and each bar represents mean ± s.e.m. of n = 3 independent experiments. Two-way ANOVA and Sidak’s multiple comparisons test was used to analyze statistical significance (*p < 0.05, **p < 0.01, ***p < 0.001, ****p < 0.0001, ns—nonsignificant). **D** Knockdown was of FKBP1A was quantified with RT-qPCR. The comparative Ct method was used with GAPDH as housekeeping gene. Unpaired t-test was used to analyze statistical significance (*p < 0.05, **p < 0.01, ***p < 0.001, ****p < 0.0001, ns—nonsignificant)

Although FK506 has been proposed to potentiate SMAD activation mainly via binding and releasing FKBP12 from the intracellular domain of the type I receptor, reports have also pointed to possible involvement of other FKBPs [43]. For instance, FKBP12.6, encoded by *FKBP1B*, is a close paralog to FKBP12 and can associate with ALK2 in a similar way as FKBP12 [44]. However, FKBP12.6 (*FKBP1B* mRNA) is not expressed in myeloma cells and therefore not relevant for these experiments (Additional file 2: Fig. S2). Other FKBPs with relevant affinities to FK506 are FKBP13 (FKBP2) (Ki = 302 nM), FKBP51 (FKBP5) (Ki = 406 nM) and FKBP52 (FKBP4) (Ki = 56 nM) [36], all of which are expressed in myeloma cell lines. To get an overview of the most likely FKBPs involved in potentiation of BMP activity by FK506 we used a panel of FKBP-binders (Table 2) and included CsA, another calcineurin inhibitor [12], as a negative control. The compounds were used to treat INA-6 BRE-luc reporter cells and to measure relative SMAD1/5/8-activity when combined with BMP6. As expected, CsA did not potentiate the BMP6 effect (Fig. 2A). Rapamycin (RAP) and compound 19 are defined as PAN-specific FKBP-binders and were approximately as potent as FK506. The FKBP-binder Selective Antagonist of FKBP51 by induced fit 1 (SAFit1) was developed to avoid binding to FKBP52, and it also has a much lower affinity to FKBP12 than for FKBP51 [45]. SAFit1 did not potentiate BMP6 activity with the doses used here. Lastly, compound 19^(S)-Me^ is a very potent FKBP binder that binds FKBP12 in the picomolar range, and FKBP51/FKBP52 in the nanomolar range [37], and potentiated BMP6 activity similarly as FK506 and its analog compound 19. Additional doses are shown for compounds FK506, 19, and 19^(S)-Me^ (Additional file 3: Fig. S3). Taken together, our results support that FKBP12 is the FKBP that regulates ALK2 activity in these cells.

**Table 2:** Affinities of FKBP-binding compounds

FKBP-protein	FK506 (nM)	RAP (nM)	SAFit1 (nM)	19 (nM)	19^(S)-Me^ (nM)
FKBP12	0.55	0.6	184.8	0.65	0.33
FKBP12.6	2.17	0.4	50.1	1.23*	nd
FKBP13	301.9	7.2	614.8	60.5*	nd
FKBP51	405.5	3.7	21.7	12	2.9
FKBP52	56.4	4.2	nb	11	3.2

Affinities for selected FKBP compounds measured by a competitive fluorescence polarization were published earlier and are shown as inhibitory constants (Ki) in nM [36, 37, 46], *; values from [36], RAP; rapamycin, nb; not binding, nd; not determined

We decided to look closer into FKBP12’s specific role by knocking down the expression of the gene encoding FKBP12, *FKBP1A*. When transfecting cells with siRNA targeting *FKBP1A* we saw a significant increase in SMAD1/5/8 activation compared with control cells when BMP6 was added (Fig. 2B, C). The knockdown was significant as shown by protein levels (Fig. 2B) and mRNA expression (Fig. 2D). These results show that FKBP12 plays an important role in regulation of SMAD-activity in this cell line. They also indicate that FK506 binding to FKBP12, and not to other FKBPs, is the main cause of the increased ligand induced SMAD1/5/8 activation, as removing FKBP12 had a similar effect as adding FK506.

### FKBP12 regulated ALK2 ligand specificity

**Fig. 3:**
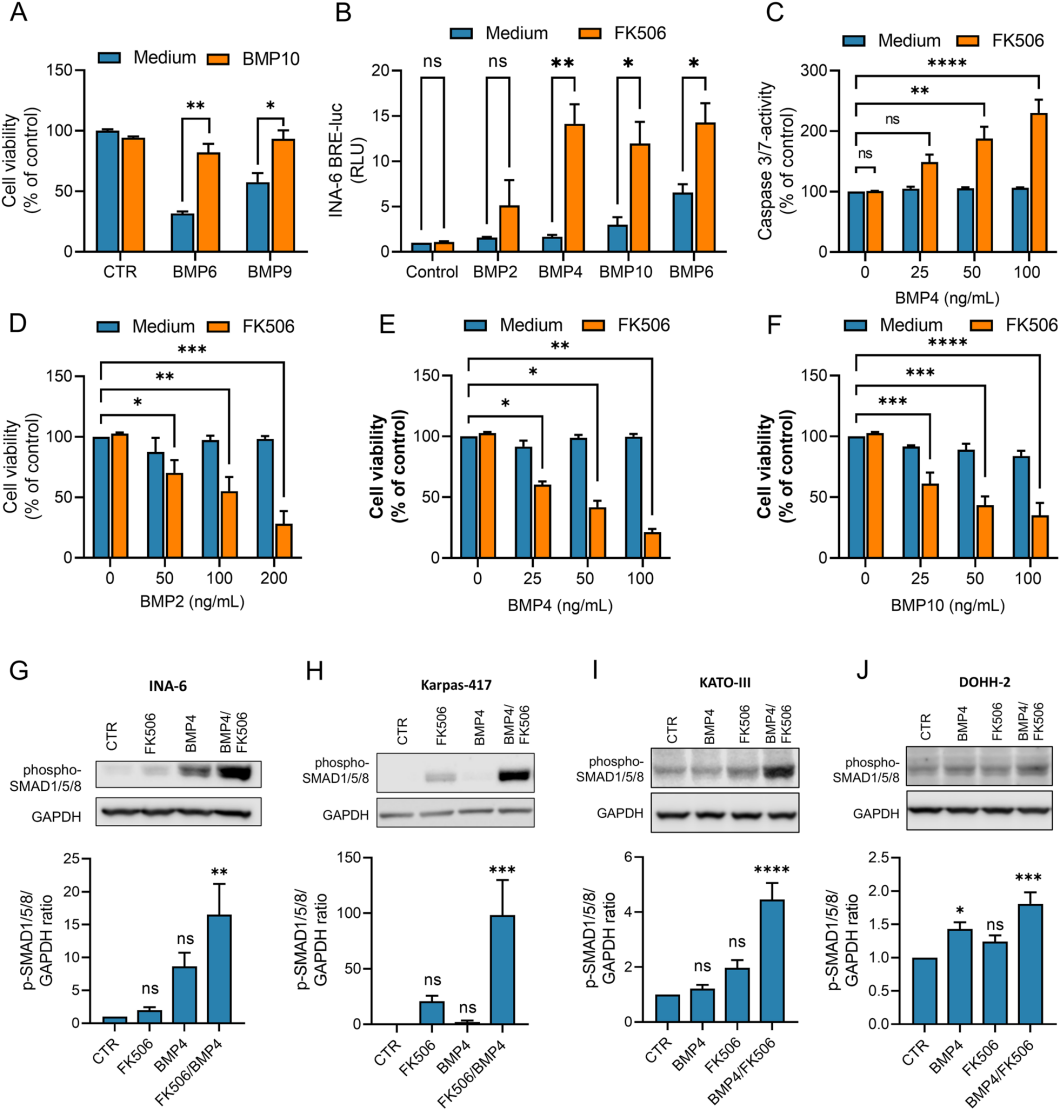
FKBP12 regulated ALK2 ligand specificity. **A** INA-6 cells were treated with BMP6 (20 ng/mL) or BMP9 (0.1 ng/mL) in the presence of BMP10 (125 ng/mL) for 72 h. The relative cell viabilities were measured with CellTiter Glo. **B** INA-6 BRE-luc cells treated with BMP2 (200 ng/mL), BMP4 (100 ng/mL), BMP10 (100 ng/mL) and BMP6 (7.5 ng/mL), with or without FK506 (100 nM) for 18 h. The results are shown as relative luciferase units (RLU) and each bar represents mean ± s.e.m. of n = 3 independent experiments. Two-way ANOVA and Sidak’s multiple comparisons test was used to analyze statistical significance (*p < 0.05, **p < 0.01, ***p < 0.001, ****p < 0.0001, ns—nonsignificant). **C** To assess downstream effect on apoptosis, INA-6 cells were treated with increasing doses of BMP4 with or without FK506 (100 nM) for 48 h before Caspase-Glo 3/7 Reagent was used to determine caspase 3/7 activity. Two-way ANOVA and Tukey’s multiple comparisons test was used to analyze statistical significance (*p < 0.05, **p < 0.01, ***p < 0.001, ****p < 0.0001, ns—nonsignificant). Then, INA-6 cells were treated with increasing concentrations of BMP2 (**D**), BMP4 (**E**), or BMP10 (**F**), with or without FK506 (100 nM) for 72 h. The relative cell viabilities were measured with CellTiter Glo. Two-way ANOVA and Tukey’s multiple comparisons test was used to analyze statistical significance (*p < 0.05, **p < 0.01, ***p < 0.001, ****p < 0.0001, ns—nonsignificant). Phosphorylated SMAD1/5/8 was measured using western blot on INA-6 (**G**) and KARPAS-417 cells (**H**) treated with BMP4 (5 ng/mL) and FK506 (100 nM) for 18 h. Phosphorylated SMAD1/5/8 was measured using western blot on KATO-III (**I**) and DOHH-2 (**J**) after 2 h stimulation with BMP4 (50 ng/mL) and FK506 (250 nM). All graphs are plotted relative to medium control and each bar represents mean ± s.e.m. of n = 3 independent experiments. Two-way ANOVA and Sidak’s multiple comparisons test was used to analyze statistical significans significance (*p < 0.05, **p < 0.01, ***p < 0.001, ****p < 0.0001, ns—nonsignificant)

ALK2 is the only BMP type I receptor expressed in sufficient levels in the INA-6 cell line for BMPs to induce apoptosis, and even high concentrations of the ALK3 ligand BMP4 did not induce apoptosis in previous studies [28, 30, 34]. We and others have previously shown that TGF-β family ligands compete for binding to receptors [3, 5, 7]. BMP10, is a potential ALK3 activating ligand, which has a strong affinity for the type II receptors ACVR2A and ACVR2B [3, 7]. Here, even high levels of BMP10 did not induce apoptosis on its own, but rather antagonised BMP6- and BMP9-induced apoptosis, likely by competing for type II receptors in the INA-6 cells (Fig. 3A). We wanted to see what happened if the ALK3 ligands BMP2, BMP4, and BMP10 were combined with FK506 in these cells. Interestingly, all three ligands were able to activate SMADs as measured by increased BRE-luc luciferase activity when combined with FK506 (Fig. 3B). Moreover, BMP4 dose-dependently activated caspase 3/7 when combined with FK506 (Fig. 3C), and we saw a dose-dependent reduction in INA-6 cell viability in the presence of FK506 (Fig. 3D–F). Similar results were seen for BMP4-induced SMAD1/5/8 phosphorylation in INA-6 and KARPAS-417, another cell line lacking ALK3 but not ALK2 (Keats Laboratory database, https://www.keatslab.org) (Fig. 3G, H). We were curious if this effect could be seen in other cell types that express ALK2, but low/absent amounts of ALK3. Using the Broad Institute Database, we chose the stomach cancer cell line KATO-III and the B-cell lymphoma cell line DOHH-2. We measured the expression levels of ALK2, ALK3, and also ALK6, using RT-qPCR and found the ALK3 and ALK6 levels to be very low compared with ALK2 for all four cell lines (Additional file 4: Fig. S4). The cells were treated with BMP4 with and without FK506 and SMAD1/5/8 activation was measured (Fig. 3I, J). Like we observed for INA-6 and KARPAS-417, also in these cell lines BMP4 induces signaling poorly but is enhanced when FK506 is co-supplied. We conclude that under the conditions shown here, FK506 can regulate both the activity and the specificity of BMP-induced SMAD1/5/8 activation via ALK2.

### BMP2, BMP4 and BMP10 require BMPR2 for ALK2 mediated signaling

**Fig. 4:**
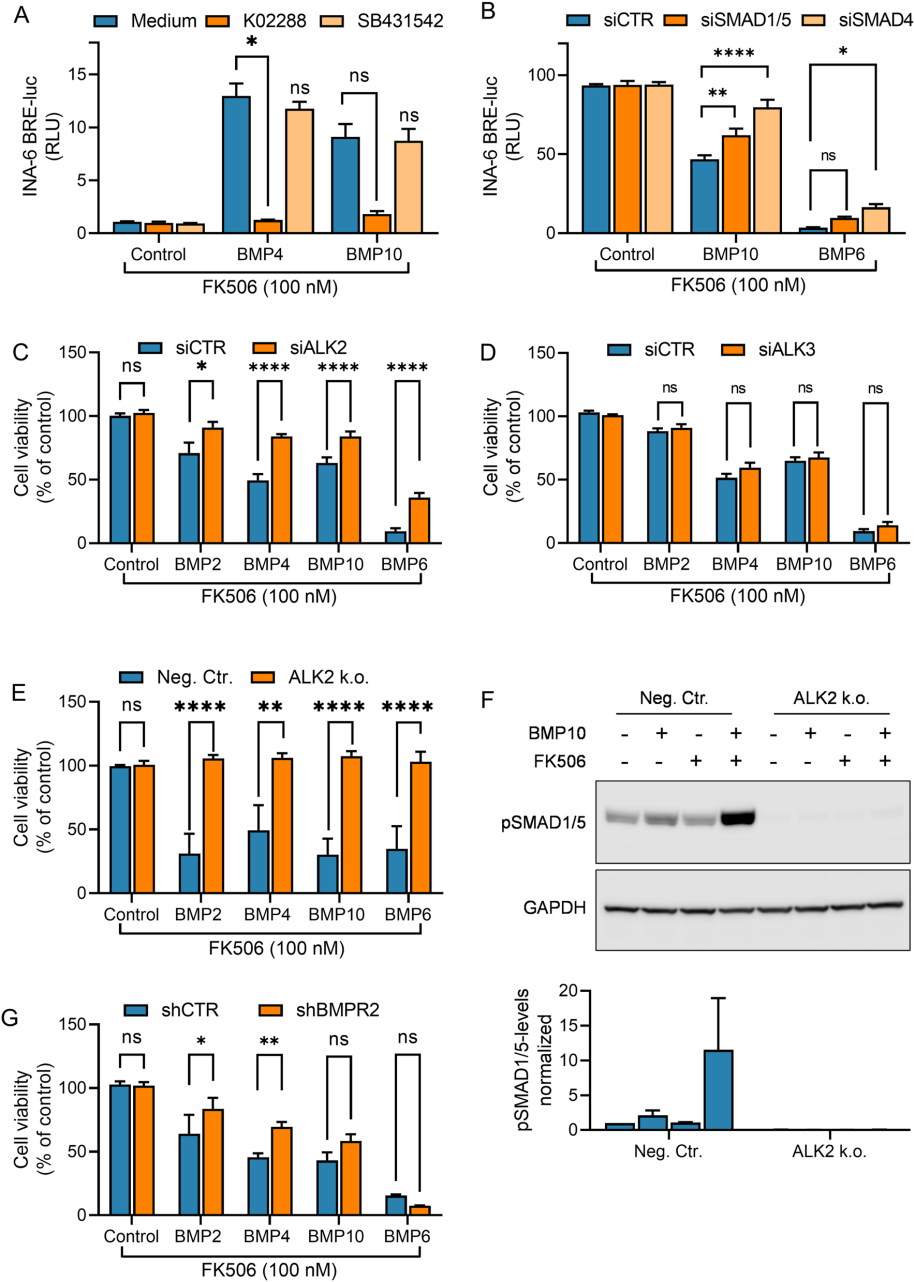
BMP2, BMP4 and BMP10 activated SMAD1/5/8 via ALK2 and BMPR2 when combined with FK506. **A** INA-6 BRE-luc cells, all in the presence of FK506 (100 nM), were treated for 18 h with BMP4 (100 ng/mL), BMP10 (100 ng/mL), with or without ALK2 kinase inhibitor K02288 (100 nM) or ALK4/5/7 kinase inhibitor SB431542 (2 µM). Luciferase substrate was added, and relative luciferase units (RLU) were measured. **B**-**D** INA-6 cells were transfected with the indicated siRNAs and treated for 48 h with BMP2 (100 ng/mL, only C and D), BMP4 (100 ng/mL, only C and D), BMP10 (100 ng/mL), or BMP6 (7.5 ng/mL), in the presence of FK506 (100 nM). The relative cell viabilities were measured with CellTiter Glo. **E** INA-6 control cells and ALK2 knock out cells were treated for 72 h with BMP2 (200 ng/mL), BMP4 (100 ng/mL), BMP10 (100 ng/mL), or BMP6 (7.5 ng/mL), in the presence of FK506 (100 nM). **F** INA-6 control cells and ALK2 knock out cells were treated with BMP10 (100 ng/mL), with or without FK506 (100 nM), for 2 h and subjected to western blotting. The blot is representative of n = 3 independent experiments (upper part). The bands from all independent experiments were quantified to determine the expression of phospho-SMAD1/5 and were normalized to GAPDH levels (lower part). **G** INA-6 cells stably transduced with BMPR2 or CTR shRNAs were treated for 48 h with BMP2 (100 ng/mL), BMP4 (100 ng/mL), BMP10 (100 ng/mL), or BMP6 (4 ng/mL), in the presence of FK506 (100 nM). The relative cell viability was measured with CellTiter Glo. All graphs are plotted relative to medium control and each bar represents mean ± s.e.m. of n = 3 independent experiments. Two-way ANOVA and Tukey’s multiple comparisons test was used to analyze statistical significance (*p < 0.05, **p < 0.01, ***p < 0.001, ****p < 0.0001, ns—nonsignificant)

Next, we wanted to characterize all components of the functional signaling receptor complex that had gained ability to induce SMAD1/5/8 activation in INA-6 cells. This is important to clarify since BMP2 and BMP4 mainly signal via ALK3 and BMPR2 [47]. BMP10 on the other hand is a high-affinity ligand for ALK1, but may also signal via ALK3, primarily combined with BMPR2, or via ALK2 combined with BMPR2 and ACVR2A [48, 49]. We first analyzed how BMP type I receptor kinase inhibitors selective for ALK1/2 (K02288) [41] and ALK4/5/7 (SB431542) [50] affected SMAD1/5/8-activation, and as expected only K02288 blunted the induction of SMAD-activity of BMP4 and BMP10 in combination with FK506 (Fig. 4A). To show more specifically the role of SMAD1/5-SMAD4 in the reduced cell viability when combining BMPs and FK506, we used siRNA targeting the combination of SMAD1 and SMAD5 or only SMAD4. Depletion of either SMAD1/5 or SMAD4 counteracted the reduced cell viability seen with BMP10 or BMP6 with FK506 when compared with non-targeting siRNA, while SMAD4 depletion was more potent (Fig. 4B, Additional file 5: Fig. S5A-C). Then we used siRNA to knock down the expression of ALK2 (*ACVR1*) in INA-6 cells. All three ligands less potently induced apoptosis in the cells treated with ALK2 siRNA and FK506 (Fig. 4C, Additional file 5: Fig. S5A), whereas without FK506 none of the ligands affected cell viability. For comparison, Fig. 4A–E, G are shown with the corresponding results without FK506 in Additional file 6: Fig. S6. In contrast, we saw no difference in cells treated with ALK3 (*BMPR1A*) siRNA compared to siCTR cells (Fig. 4D, Additional file 5: Fig. S5D-EA), further supporting that ALK2 is the receptor responsible for this functional outcome. We also generated ALK2 (*ACVR1*) knock-out cells using CRISPR/Cas9 technology. The knock-out was confirmed by sequencing as described in the Methods section. As expected, the results show even more clearly that ALK2 is necessary for BMP2, BMP4 and BMP10 to signal in these cells as shown by maintained cell viability in the ALK2 k.o. cells compared to the negative control cells (Fig. 4E). Likewise, the SMAD1/5 activation by BMP10 with FK506 was blunted in the ALK2 k.o. cells (Fig. 4F). Finally, we previously reported that the more common ALK2 ligands, such as BMP6, BMP9, and activin B, prefer ACVR2A or ACVR2B over BMPR2 as type II receptor in these cells and that their activity is increased when BMPR2 is knocked down [34]. Interestingly, this was not the case for BMP2, BMP4 and BMP10 that had gained activity via ALK2 by FK506 co-treatment. These three ligands were dependent on BMPR2 for full signaling activity, as shown by a lower reduction in cell viability in cells with shRNA targeting BMPR2, and as expected this contrasted with the effect of BMP6 (Fig. 4G, Additional file 5: Fig. S5F). For comparison, Fig. 4A-E, G are shown with the corresponding results without FK506 in Additional file 6: Fig. S6. In summary, we show that the ligands that usually signal via ALK3 can gain ability to signal via ALK2, but this is dependent on both the removal of FKBP12 and the presence of BMPR2.

## Discussion

Multiple myeloma cells are sensitive to BMP-induced apoptosis and growth arrest [26–30]. Increasing BMP activity in bone marrow to reduce tumor growth and promote bone formation could thus be beneficial for myeloma patients. Here we show that the immunosuppressive drug FK506 potentiated SMAD1/5/8-activation via the TGF-β/BMP type I receptor ALK2, and thereby increased myeloma cell death. FK506 binds many different FKBPs, but FKBP12 is the main FKBP to bind TGF-β type I receptors and regulate their activity [15]. FKBP12, like many other FKBPs have *cis*–*trans* peptidyl-prolyl isomerase (PPIase) activity and bind TGF-β type I receptors by a protein–protein interaction that uses the PPIase active site but does likely not involve any *cis–trans* isomerization step [15]. The close analog to FKBP12, FKBP12.6, can also bind to TGF-β type I receptors in a similar way but as we show here, this FKBP is not expressed in myeloma cells [44]. When we knocked down the gene encoding FKBP12, *FKBP1A,* we found a similar potentiation of BMP-induced SMAD-activation as we saw with FK506 cotreatment. This supported our hypothesis of FKBP12 having an important role in potentiation of SMAD1/5/8 activation by FK506.

We also compared the effects of FK506 with that of other FKBP-binding compounds with varying affinity profiles. One of the compounds, RAP, binds most of the FKBPs, including FKBP12, with very high affinities (Table 2). Like FK506, RAP forms an immunosuppressive complex when bound to FKBP12, but the mechanism is via inhibition of mTOR rather than inhibition of calcineurin [15]. In our experiments RAP was at least as strong as FK506 in potentiating BMP6-mediated SMAD-activity. This contrasts to a study where RAP was found to be less efficient than FK506 in potentiating BMP4 activity in C2C12 murine myoblast cells and pulmonary arterial endothelial cells (PAEC) [43]. In that study, the authors found that FK506’s effect on BMP signaling also required the inhibition of calcineurin for full effect. They further suggested that the poorer activity seen with RAP compared with FK506 was partly due to lack of calcineurin inhibition and partly because RAP only engaged with ALK2, which is not thought to be a BMP4 receptor. Other studies using non-immunosuppressive FKBP-targeting drugs have found that potentiation of BMP did not require inhibition of calcineurin [51, 52]. Our results suggest that, at least with our experimental conditions, the sequestration or removal of FKBP12 is sufficient to potentiate BMP-induced SMAD1/5-activation.

It has been suggested that FK506 can induce SMAD activity independently of ligand [15, 53, 54]. Due to the potency of FK506, it is possible that some cells produce sufficient endogenous ligand to enable FK506-mediated SMAD activation. In some studies the natural antagonist of BMPs, noggin, was added to rule out the presence of BMPs [53, 54], however noggin binds only selected ligands and will not fully inhibit all BMPs that might be present [55]. We performed most of our experiments in the presence of 2% human serum to keep the cells alive. SMAD1/5/8 activation was seen with FK506 without BMPs added in some experiments (Fig. 2F, G). However, since serum contains functional BMPs [56], we chose to perform the BRE-luc reporter assays in serum-free medium and did not see any SMAD activity beyond control levels by just adding FK506 or any other FKBP-binding compounds. Similar results have been seen by others, such as in assays investigating FK506’s effect on the *ACVR1* R206H FOP mutation [6]. We therefore suggest that FK506 do not activate SMAD1/5/8 if no BMPs or activins are present in the culture, either via serum or through endogenous production.

A question that remains is why the cell lines responded differently to FKBP12 removal, as in some cell lines we saw no potentiating effect of FK506 cotreatment with BMPs. There are several possibilities to explain this: First, to compare FK506-effects we used a functional assay (cell viability) and did not measure SMAD1/5/8 activation for all cell lines. It is possible that some of the tested cell lines are resistant to BMP-induced apoptosis even if the SMAD1/5/8 pathway is activated. To fully compare the effect of FK506 on BMP-signaling between cell lines we would therefore have to compare the phosphorylation of SMAD1/5/8 in all the cell lines. Secondly, we speculate that the differences between cell lines in response to FK506 could be due to different abundance of FKBP12 and other FKBPs that will bind to FK506. However, this will be investigated in more detail in future studies.

Most surprisingly we identified here a switch in ligand specificity as BMP2, BMP4, and BMP10, ligands that usually signal via ALK3 in myeloma cells, gained the ability to signal via ALK2 in the presence of FK506. For this, we used a cell line in which the mentioned ALK3 ligands have no effect on cell viability alone, but when combined with FK506 all three ligands dose-dependently induced apoptosis. Since INA-6 cells can express low levels of ALK3 we wanted to clarify which type I receptor was mediating the gained activity. Using siRNA, we found that ALK3 knockdown made no difference on SMAD activation. Instead, we found that ALK2 was necessary for BMP2-, BMP4-, and BMP10-induced activation of SMAD1/5/8 and consequently apoptosis in these cells, and that the type II receptor BMPR2 was needed for full signal activity. This contrasts with the common ALK2 ligands such as BMP6, BMP9 and activins, as we have shown that BMPR2 inhibits their activity probably by competing with ACVR2A and/or ACVR2B for complex formation with ALK2 [34, 42]. We saw the same gain of activity in a stomach cancer cell line and a B cell lymphoma cell line, both expressing similar levels of type I receptors as INA-6 and KARPAS-417, giving our results a relevance beyond multiple myeloma.

Competition between high- and low-affinity ligands for receptor binding is context dependent, influences signaling outcome and has important physiological implications [3, 57, 58]. Loss of BMPR2 cause a context-dependent gain-of-activity and we found that ALK2 ligands were more efficient when we knocked down *BMPR2* [34, 59–62]. This suggests that ALK2 is a limiting factor in these cells and that there is a competition between BMPR2 and ACVR2A/B for complex formation with ALK2. Here, ALK3 ligands were non-signaling in cells only expressing ALK2 as type I receptor. BMP9 and BMP10 are both high-affinity receptors for the endothelial type I receptor ALK1 [48, 63], but can signal via other type I receptors in cell types where ALK1 is absent (e.g., BMP9-ALK2 and BMP10-ALK3/ALK2) [3]. BMP10 has previously been shown to compete with BMP2, BMP7, and activin A for type II receptor binding [7]. The recently solved structure of the extracellular domain of BMPR2 in complex with BMP10 indicates a relatively unstable and low affinity interaction [64]. Still, BMP10 is the ligand that binds with highest affinity to BMPR2 of the three ALK3 ligands tested here [7]. This could explain why we found that BMP10 competed with BMP6 and BMP9 in ALK2 expressing cells and antagonized their activity. The endogenous type II receptor(s) involved in this is not known, but we speculate that a combination of BMP10 with BMPR2-ALK2 could form NSCs as has been described for activin A-ALK2 [5–7, 62, 65], keeping ALK2-ACVR2A/B unavailable for its natural activating ligands.

In pulmonary arterial hypertension (PAH), *BMPR2* loss-of-function mutations are involved in the pathogenesis of many patients [66], supporting the idea of a protective role of BMP activity [67]. Mutations in *GDF2* (encoding BMP9) and *BMP10* have also been described in PAH, but the exact roles and how BMP9/BMP10 are affected by BMPR2 in PAH are still unresolved [68, 69]. The ability of BMP10 to bind BMPR2 is affected differently by different *BMPR2* mutations found in PAH [70].We here speculate that mutations in BMPR2 in some cases could prevent formation of potential NSCs, leading to changed signaling capacities. We find it very interesting that BMP10 under given circumstances can inhibit activity of its related ligand BMP9, underscoring the complexity of TGF-β signaling regulation. Of note, BMP9-BMP10 heterodimers were found to be responsible for the main biological activity of these ligands in plasma [71], but it remains to be seen how such heterodimers act when bound to ALK2-BMPR2 receptor complexes.

**Fig. 5:**
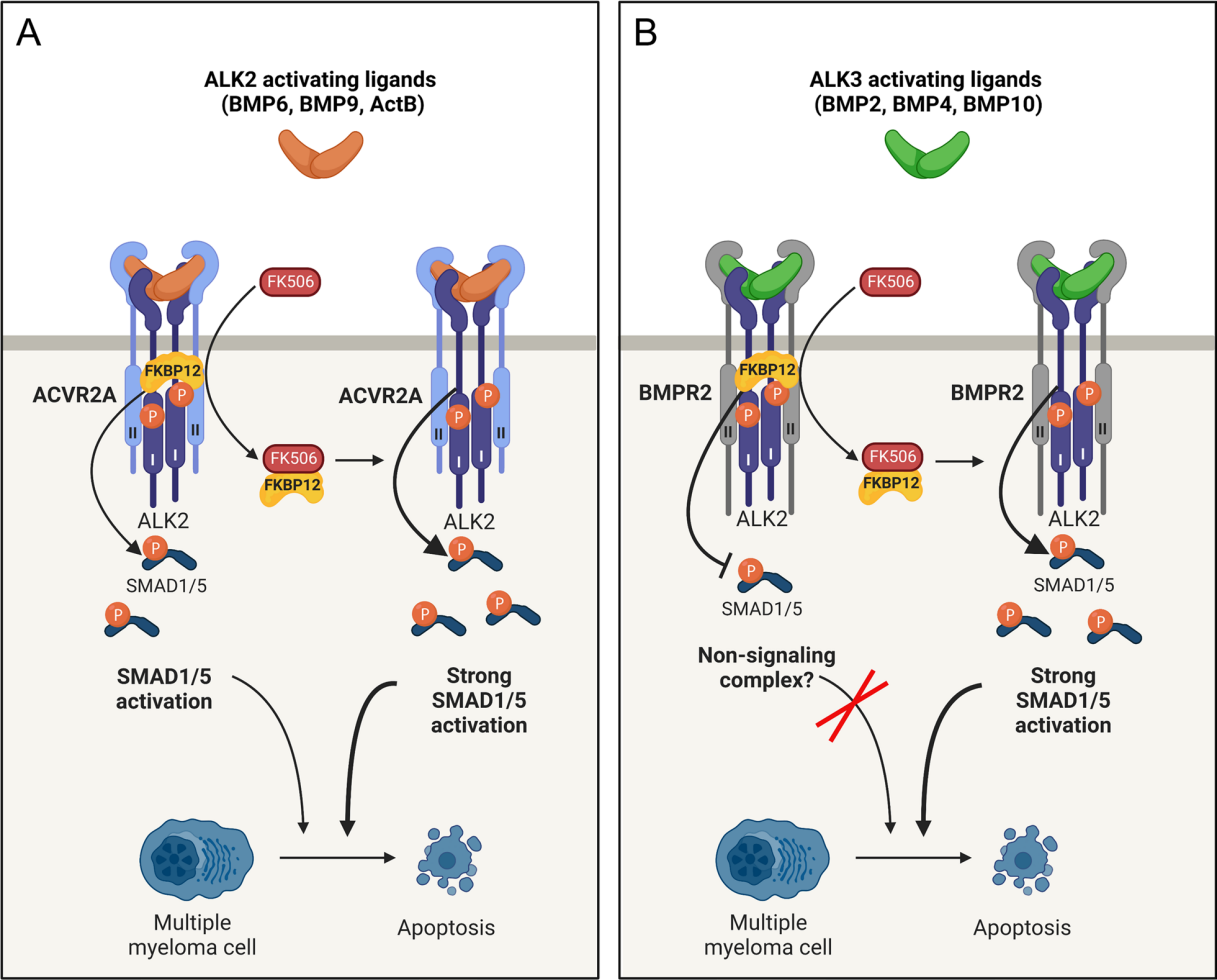
Proposed regulation of ligand dependent ALK2 activity in multiple myeloma cells. **A** ALK2 ligands such as BMP6, BMP9, and activin B signal via ALK2 preferably in complex with ACVR2A or ACVR2B. Activin A also binds to ALK2:ACVR2 but may either form a non-signaling complex (NSC) or an active signaling complex depending on the context. Addition of FK506 removes FKBP12 from the activation domain of ALK2 leading to increased ligand-induced SMAD1/5-activation. SMAD1/5-activation leads to myeloma cell apoptosis. **B** ALK3 ligands such as BMP2, BMP4, and BMP10, do not activate SMAD1/5 via ALK2 even in the presence of their preferred type II receptor BMPR2. It is unclear if the ligand can form an NSC with the receptors or not. Addition of FK506 removes FKBP12 from the activation domain of ALK2 leading to a dose-dependent ligand-induced SMAD1/5-activation and myeloma cell apoptosis. The figure was made with Biorender.com

It is puzzling how ALK3 ligands gain ability to signal via ALK2 when FKBP12 is removed. Since there was also a switch in type II receptor dependency when compared to the common ALK2 ligands, it is tempting to speculate that FKBP12 plays a critical role in determining which type II receptor can be part of the active complex. The phosphorylation of the GS domain by type II receptors upon ligand stimulation cause a release of FKBP12 and activation of the type I receptor kinases [18, 19]. If ligand-bound BMPR2, unlike ACVR2A/B, would be unable to phosphorylate ALK2’s GS domain and the subsequent release of FKBP12 from ALK2, that could explain why adding FK506 would support signaling as it removes FKBP12 and enables BMPR2 only then to phosphorylate ALK2. A proposed model of how ligand dependent ALK2 activity is regulated in multiple myeloma cells is shown (Fig. 5).

We here confirm that FK506 is a drug that clearly potentiates BMP activity. Several multiple myeloma patients have in recent years received kidney transplant as part of their treatment and 67% of the patients received tacrolimus as part of the immunosuppressive regimen following the transplant [72]. Studies have shown that these patients have an overall survival that almost reach the same as patients receiving the same myeloma treatment without the need of a kidney transplant, suggesting that these patients may have benefited from the tacrolimus treatment [72]. The development of cancers in general following transplantation is quite common [73]. Some studies have found a trend of lower risk of getting multiple myeloma after a kidney transplant, compared with the general population [74, 75], whereas other studies found a higher incident of multiple myeloma following kidney transplantation [76, 77]. Overall, it seems that giving tacrolimus to patients is safe, but that the patients might have an increased rate of infection. Therefore, it would be better to use less immunosuppressive FKBP12-targeting drug variants for cancer patients. Several non-immunosuppressive FKBP-binders are in development [51, 54, 78, 79]. The challenges going into a clinical setting involve the drugs’ bioactivity and -distribution, delivery with some degree of specificity to target cells, and avoiding off-target effects.

## Conclusions

Regulation of ALK2 activity by FKBP12 varies between cells and between different ligand-receptor combinations. We here show that FKBP12 is an important regulator of ligand dependent ALK2 activity in multiple myeloma cells and that adding FK506 in vitro strongly potentiates ALK2 activity, partly by switching a possible NSC into an active signaling complex. We propose that targeting FKBP12 with a suitable non-immunosuppressive compound could be a possible novel treatment option in cases where increased BMP activity is wanted.

## Supplementary Information

**Additional file 1: Figure S1**. Supporting data to Fig. 1. A. Example dot plots for apoptosis assay in Fig. 1C. B. Densitometric analysis of phospho-SMAD1/5 relative to GAPDH levels in Fig. 1D. C. Densitometric analysis of phospho-SMAD2/3 relative to GAPDH levels in Fig. 1D.

**Additional file 2: Figure S2.** Supporting data to Fig. 2. Relative amount of FKBP gene transcripts in 66 human myeloma cell lines.

**Additional File 3: Figure S3.** Supporting data to Fig. 2. Potentiation of BMP6 activity with FKBP-binding compounds.

**Additional File 4: Figure S4.** Supporting data to Fig. 3. Expression levels of ALK2 (ACVR1), ALK3 (BMPR1A), and ALK6 (BMPR1B) in (A) INA-6, (B) KARPAS-417, (C) KATO-III, and (D) DOHH-2 using RT-qPCR and the comparative Ct method with GAPDH as housekeeping gene.

**Additional File 5: Figure S5.** Supporting data to Fig. 4. Verification of knockdown in INA-6 cells. A-C Knockdown of SMAD1, SMAD5, and SMAD4 after use of SMAD1/5 and SMAD4 siRNAs. D, E. Knockdown after use of ALK2 (ACVR1) and ALK3 (BMPR1A) siRNAs. F. Knockdown of BMPR2 in INA-6 shBMPR2 cells.

**Additional File 6: Figure S6.** Supporting data to Fig. 4. Experiments from Fig. 4A-E, G, shown without and with FK506.

## Data Availability

Not applicable.

## Abbreviations

ALK: Activin receptor-like kinase
BMP: Bone morphogenetic protein
FKBP: FK506 binding protein
GDF: Growth differentiation factor
ID: Inhibitor of DNA-binding protein
NSC: Non-signaling complex
PPiase: Peptidyl-prolyl isomerase
TGF-β: Transforming growth factor-β
