# Supplementary material for: FKBP12 is a major regulator of ALK2 activity in multiple myeloma cells
Source: Cell Commun Signal. 2023 Jan 30;21:25. doi: 10.1186/s12964-022-01033-9 (PMC9885706; doi:10.1186/s12964-022-01033-9)
Supplement: Supplementary file 2 — Additional file 1: Figure S1. Supporting data to Fig. 1. A. Example dot plots for apoptosis assay in Fig. 1C. B. Densitometric analysis of phospho-SMAD1/5 relative to GAPDH levels in Fig. 1D. C. Densitometric analysis of phospho-SMAD2/3 relative to GAPDH levels in Fig. 1D. [file 12964_2022_1033_MOESM2_ESM.docx]

Additional File 1

A


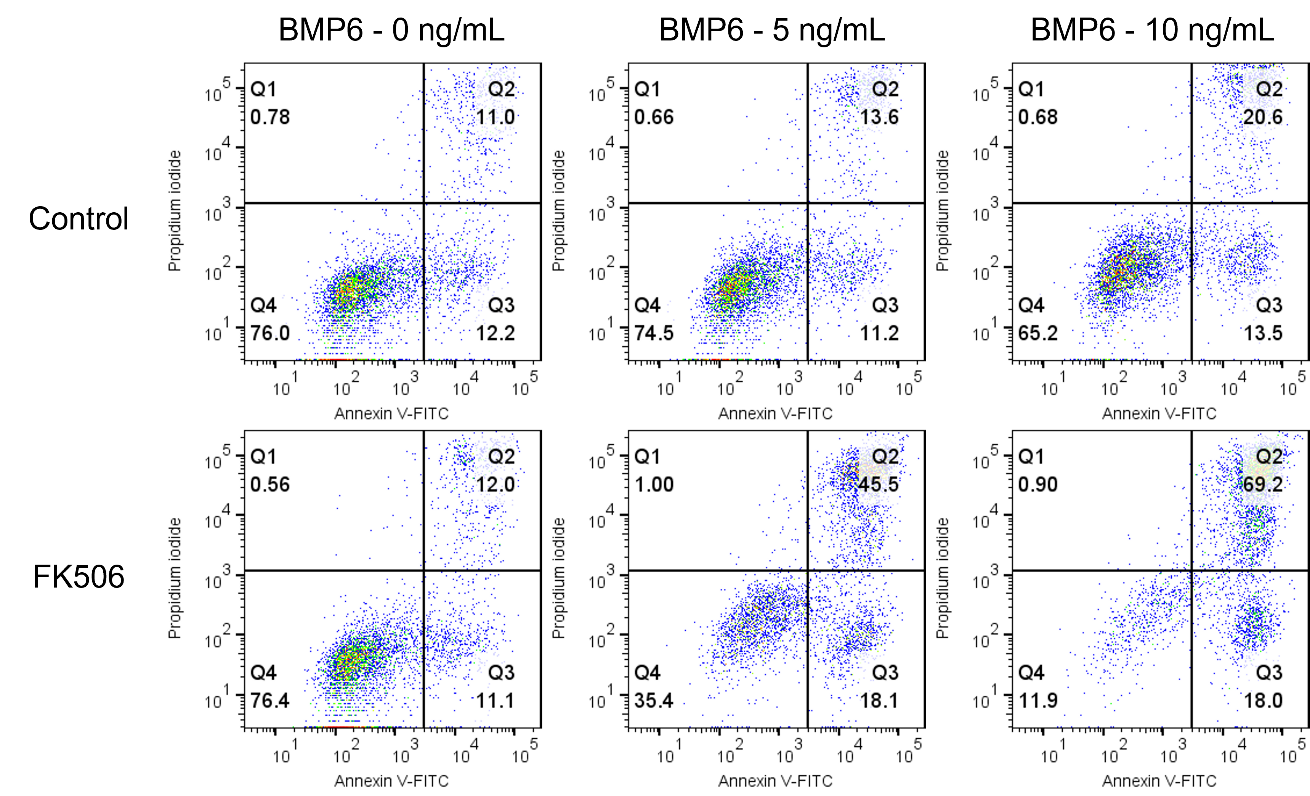


B C

**Figure S1. Supporting data to Figure 1.** A. Example of plots from the apoptosis assay showing annexin V-FITC versus propidium iodide staining. Cells that were double negative were considered viable and plotted in Fig. 1C. B, C. Related to Fig. 1D. Densitometric analysis of phospho-SMAD1/5 (B) or SMAD2/3 (C) normalized to GAPDH levels. Two-way ANOVA and Sidak’s multiple comparisons test was used to analyze statistical significance (* p<0.05, ** p<0.01, *** p<0.001, ns – nonsignificant).
