# Supplementary material for: FKBP12 is a major regulator of ALK2 activity in multiple myeloma cells
Source: Cell Commun Signal. 2023 Jan 30;21:25. doi: 10.1186/s12964-022-01033-9 (PMC9885706; doi:10.1186/s12964-022-01033-9)
Supplement: Supplementary file 3 — Additional file 2: Figure S2. Supporting data to Fig. 2. Relative amount of FKBP gene transcripts in 66 human myeloma cell lines. [file 12964_2022_1033_MOESM3_ESM.docx]

Additional File 2

**Figure S2. Relative amount of FKBP gene transcripts in 66 human myeloma cell lines.** The transcript levels of 66 human myeloma cell lines (HMCL) were obtained from Keats Laboratory database (https://www.keatslab.org) and are shown as fragments per kilobase per million (FKPM). Violin plot was created in GraphPad Prism 9.
