# Supplementary material for: FKBP12 is a major regulator of ALK2 activity in multiple myeloma cells
Source: Cell Commun Signal. 2023 Jan 30;21:25. doi: 10.1186/s12964-022-01033-9 (PMC9885706; doi:10.1186/s12964-022-01033-9)
Supplement: Supplementary file 4 — Additional File 3: Figure S3. Supporting data to Fig. 2. Potentiation of BMP6 activity with FKBP-binding compounds. [file 12964_2022_1033_MOESM4_ESM.docx]

Additional File 3

**Figure S3.** **Potentiation of BMP6 activity with FKBP-binding compounds.** INA-6 BRE-luc cells were treated for 18 h with BMP6 (7.5 ng/mL) and the indicated compounds. Luciferase substrate was added, and relative luciferase units (RLU) were measured. Results are plotted relative to BMP6 induced luciferase activity (BMP6 alone = 1) and each bar represents mean ± s.e.m. of n = 3 independent experiments. Parts of these result were published earlier (Kolos JM *et al*. Chem Sci, 2021).
