# Supplementary material for: FKBP12 is a major regulator of ALK2 activity in multiple myeloma cells
Source: Cell Commun Signal. 2023 Jan 30;21:25. doi: 10.1186/s12964-022-01033-9 (PMC9885706; doi:10.1186/s12964-022-01033-9)
Supplement: Supplementary file 5 — Additional File 4: Figure S4. Supporting data to Fig. 3. Expression levels of ALK2 (ACVR1), ALK3 (BMPR1A), and ALK6 (BMPR1B) in (A) INA-6, (B) KARPAS-417, (C) KATO-III, and (D) DOHH-2 using RT-qPCR and the comparative Ct method with GAPDH as housekeeping gene. [file 12964_2022_1033_MOESM5_ESM.docx]

Additional File 4


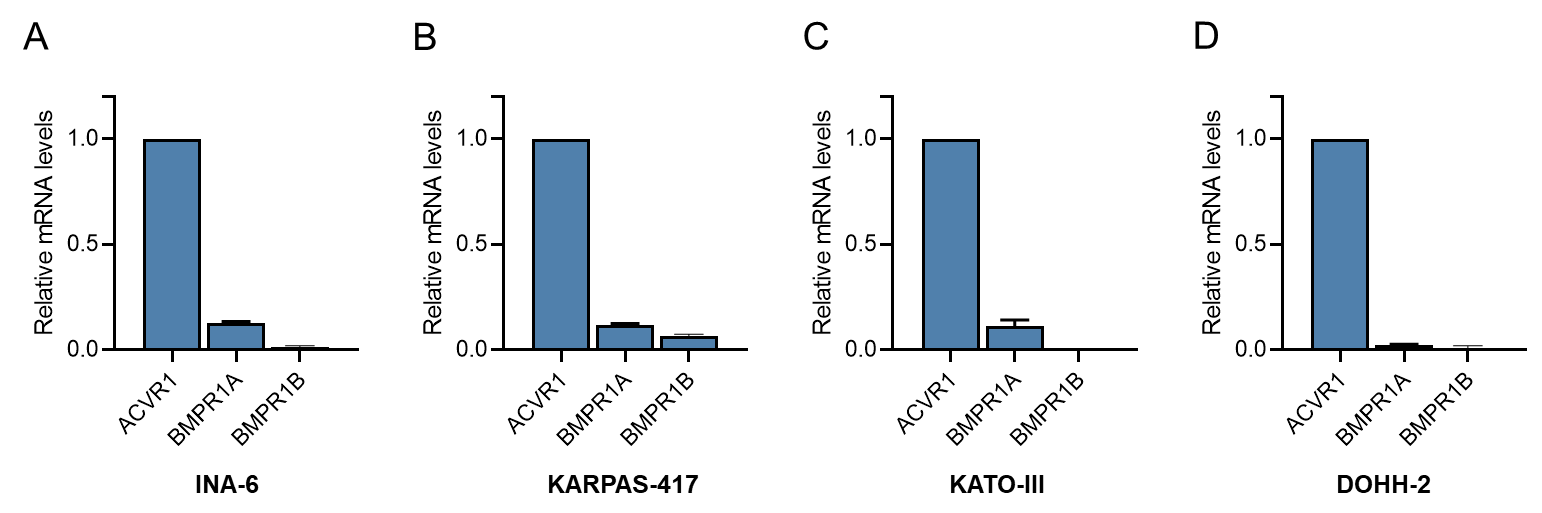


**Figure S4. BMP type I receptor gene expression.** Expression levels of ALK2 (*ACVR1*), ALK3 (*BMPR1A*), and ALK6 (*BMPR1B*) were measured in (A) INA-6, (B) KARPAS-417, (C) KATO-III, and (D) DOHH-2 using RT-qPCR and the comparative Ct method with *GAPDH* as housekeeping gene. The graphs represent mean ± s.e.m. of n=3 independent experiments.
