# Supplementary material for: FKBP12 is a major regulator of ALK2 activity in multiple myeloma cells
Source: Cell Commun Signal. 2023 Jan 30;21:25. doi: 10.1186/s12964-022-01033-9 (PMC9885706; doi:10.1186/s12964-022-01033-9)
Supplement: Supplementary file 6 — Additional File 5: Figure S5. Supporting data to Fig. 4. Verification of knockdown in INA-6 cells. A-C Knockdown of SMAD1, SMAD5, and SMAD4 after use of SMAD1/5 and SMAD4 siRNAs. D, E. Knockdown after use of ALK2 (ACVR1) and ALK3 (BMPR1A) siRNAs. F. Knockdown of BMPR2 in INA-6 shBMPR2 cells. [file 12964_2022_1033_MOESM6_ESM.docx]

Additional File 5


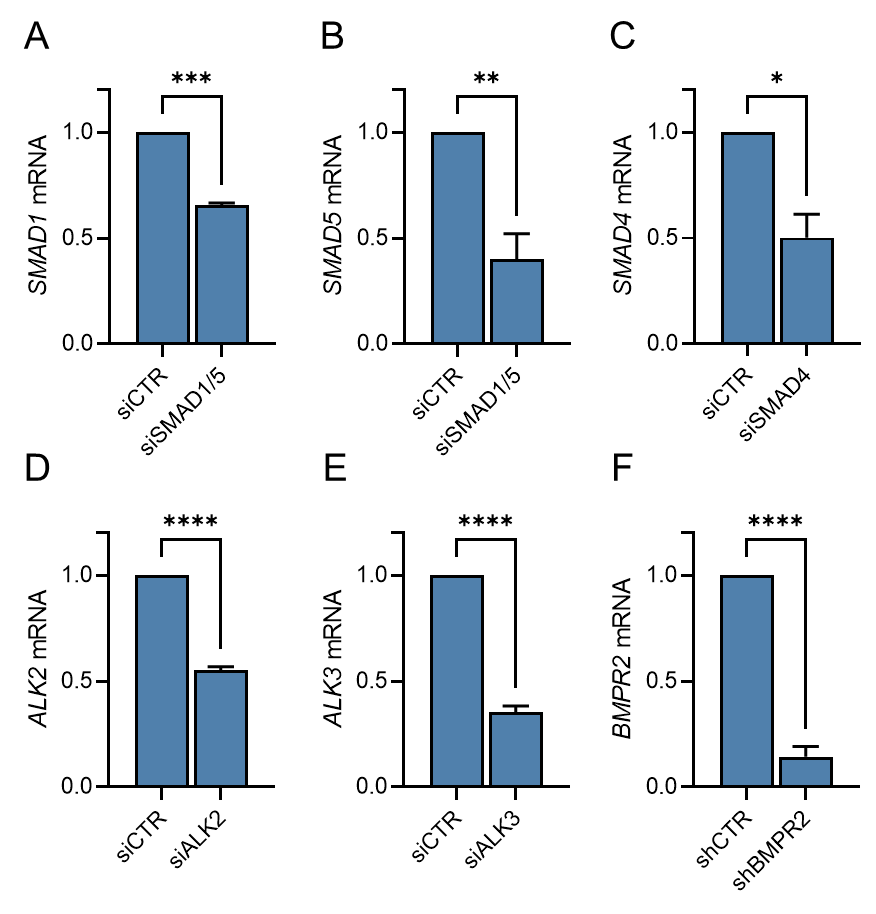


**Figure S5. Verification of knockdown in INA-6 cells.** The mRNA levels of SMAD1 (A), SMAD5 (B), and SMAD4 (C) in cells transfected with the indicated siRNA compared with cells transfected with non-targeting control siRNA. The mRNA levels of ALK2 (*ACVR1*) (D) and ALK3 (*BMPR1A*) (E) in cells transfected with siRNAs were measured and compared with cells transfected with non-targeting control siRNA. F. The mRNA levels of *BMPR2* in were measured in stably transduced INA-6 cells and compared with cells with stable expression of non-targeting shRNA. In all cases, we used RT-qPCR using the comparative Ct method and GAPDH as housekeeping gene. Unpaired t-test was used to analyze statistical significance (* p<0.05, ** p<0.01, *** p<0.001, **** p<0.0001).
