# Supplementary material for: FKBP12 is a major regulator of ALK2 activity in multiple myeloma cells
Source: Cell Commun Signal. 2023 Jan 30;21:25. doi: 10.1186/s12964-022-01033-9 (PMC9885706; doi:10.1186/s12964-022-01033-9)
Supplement: Supplementary file 7 — Additional File 6: Figure S6. Supporting data to Fig. 4. Experiments from Fig. 4A-E, G, shown without and with FK506. [file 12964_2022_1033_MOESM7_ESM.docx]

Additional File 6

A

B

C

D

E

F

**Figure S6. Experiments corresponding to Figure 4 shown without and with FK506.** A-E. Corresponding to Fig. 4A-E. F. Corresponding to Fig. 4G.
